# Correlation between estrogen receptor β expression and the curative effect of endocrine therapy in breast cancer patients

**DOI:** 10.3892/etm.2014.1634

**Published:** 2014-03-27

**Authors:** LIYING GUO, YU ZHANG, WEI ZHANG, DILIMINA YILAMU

**Affiliations:** 1 Department of Breast Cancer, Digestive and Vascular Center, The First Affiliated Hospital of Xinjiang Medical University, Urumqi, Xinjiang 830054, P.R. China; 2 Department of General Surgery, Yantai Affiliated Hospital of Binzhou Medical University, Yantai, Shandong 264100, P.R. China; 3 Department of Pathology, The First Affiliated Hospital of Xinjiang Medical University, Urumqi, Xinjiang 830054, P.R. China

**Keywords:** breast cancer, estrogen receptor β, curative effect

## Abstract

The aim of the present study was to investigate the association between the expression levels of estrogen receptor (ER)β and the curative effect of endocrine therapy in breast cancer patients. Cancer tissues were collected from 583 breast cancer patients between January 2000 and December 2010 and used for analysis. ERβ expression levels were determined using immunohistochemical staining. The Kaplan-Meier method was used for survival analysis and the log-rank test was conducted for difference analysis between survival times. In addition, Cox multivariate analysis was performed to analyze prognostic factors for breast cancer. In the immunohistochemical staining assay, a positive ERβ expression rate of <10% was defined as ERβ low expression, while >10% was defined as ERβ high expression. In patients expressing low levels of ERβ, the median tumor-free survival time of the patients who received endocrine therapy was significantly higher compared with that of the patients who did not receive endocrine therapy. By contrast, in patients with high ERβ expression levels, there was no significant difference in the median tumor-free survival time between the patients who received endocrine therapy and those who did not. In addition, compared with ERβ low expression patients, ERβ high expression patients had a significantly lower median tumor-free survival time. Furthermore, ERβ expression, human epidermal growth factor receptor 2 expression, tumor size, lymph node metastasis, postoperative chemotherapy, radiotherapy and endocrine therapy were identified to be independent prognostic factors for breast cancer. Therefore, high ERβ expression in breast cancer indicates poor prognosis for endocrine therapy.

## Introduction

Breast cancer is a hormone-dependent tumor that involves the interaction of estrogen and its specific receptors. Estrogen receptor (ER)β, as reported by Kuiper *et al* (1), was initially identified in the cDNA library of rat prostate cells and is a subtype of the ER superfamily. ERβ is known to be widely expressed in normal cells and tumor tissues of humans and rats. The expression levels of ERβ in ovarian, liver, prostate, small intestine and colorectal cancers have been reported to be associated with tumor occurrence, development and malignancy (2). Notably, ERβ is of great significance for breast cancer and ERβ expression levels in breast cancer are closely associated with the curative effect of postoperative endocrine therapy (3).

Endocrine therapy is an effective method for the treatment of estrogen-sensitive breast cancer. Esslimani-Sahla *et al* (4) hypothesized that ERβ protein levels in breast cancer are associated with the efficacy of endocrine therapy. Hopp *et al* (5) found that ERβ was highly expressed in endocrine-resistant breast cancer cells. By contrast, Borgquist *et al* (6) reported that low ERβ expression resulted in a poor prognosis of endocrine therapy. Therefore, the role of ERβ in endocrine resistance remains controversial.

In the present study, the association between ERβ expression and the efficacy of endocrine therapy in breast cancer was systematically investigated. Cancer tissues from 598 patients with breast cancer were used in the study and the expression levels of ERβ were determined by immunohistochemistry. Survival analysis was conducted between patients with ERβ low or high expression and patients who received or did not receive endocrine therapy. In addition, the prognostic factors for breast cancer were analyzed by Cox multivariate analysis.

## Materials and methods

### Clinical data

In total, 598 patients with pathologically confirmed invasive breast cancer were enrolled in the study. All individuals were diagnosed and treated in the First Affiliated Hospital of Xinjiang Medical University (Ürümqi, China) between January 2000 and December 2010. The clinical features of the patients are shown in Table I. Patients received follow-ups for 2–10 years. During the follow-up period, 15 patients were censored due to the loss of contact during the follow-up period or prior to the study cut-off point, or due to mortality from other causes.

Prior written and informed consent was obtained from every patient and the study was approved by the Ethics Review Board of Xinjiang Medical University.

### Immunohistochemistry

Breast cancer tissue specimens were fixed in 10% formaldehyde for 24 h and then embedded in paraffin. The specimens were then sliced into 3-μm sections. Following dewaxing and rehydrating in graded alcohols, sections were incubated with anti-ERβ primary antibodies. An ERβ positive sample was used as a positive control. In the negative control, the primary antibody was replaced with phosphate-buffered saline. The anti-ERβ antibodies and the working solution were purchased from Fuzhou Maixin Biotechnology Development Co., Ltd. (Fuzhou, China).

### Determination of ERβ expression levels

Cells with brown staining in the nucleus were considered ERβ positive cells. Five fields at high-magnification were randomly selected. The ERβ positive rate was the ratio of the number of ERβ positive cells to the total number of cells in each field. An ERβ positive rate <1% was defined as ERβ negative (−). A positive rate between 1 and 10% was defined as ERβ weak positive (+) and an ERβ positive rate between 10 and 50% was defined as ERβ positive (++). Finally, an ERβ positive rate >50% was defined as ERβ strong positive (+++). Cells defined ERβ (−) and (+) were considered to be ERβ low expression cells, while cells defined ERβ (++) and ERβ (+++) were considered to be ERβ high expression cells.

### Statistical analysis

SPSS statistical software, version 17.0 (SPSS, Inc., Chicago, IL, USA) was used for statistical analysis. Kaplan-Meier survival curves were constructed for survival analysis and the log-rank test was used to determine the differences in survival. Cox multivariate analysis was also performed to analyze prognostic factors. P<0.05 was considered to indicate a statistically significant difference.

## Results

### Expression of ERβ in breast cancer

**Figure 1:**
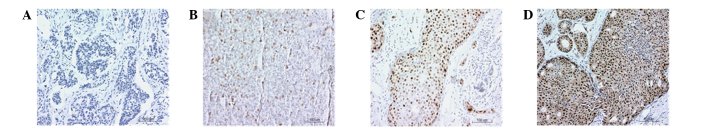
Expression analysis of ERβ in breast cancer tissues using immunohistochemistry (magnification, ×100). Representative images are shown and cells with brown staining in the nucleus were considered ERβ positive cells. Scale bar, 100 μm. (A) ERβ negative cells; (B) ERβ weak positive expression cells with a rate of <10%; (C) ERβ positive expression cells with a rate between 10 and 50%; (D) ERβ high expression cells with a rate of >50%. ER, estrogen receptor.

The expression levels of ERβ in the breast cancer tissue samples were analyzed by immunohistochemical staining. Representative results are shown in Fig. 1. Cells with brown particles in the nucleus were ERβ positive cells. There were no cells with brown staining visible in Fig. 1A, indicating that ERβ expression was negative. However, in Fig. 1B–D, certain cells were positively stained, indicating a positive expression of ERβ. Cells in which the expression of ERβ was indicated were counted and the positive expression rate was calculated. Weak expression of ERβ with a positive rate of <10% is shown in Fig. 1B. Positive expression of ERβ with a positive rate between 10 and 50% is demonstrated in Fig. 1C and high expression of ERβ with a positive rate >50% is shown in Fig. 1D. Cells that were classified as ERβ (−) or (+) were defined as ERβ low expression cells, while cells that were classified as ERβ (++) or (+++) were defined as ERβ high expression cells.

### Median tumor-free survival time is longer in patients with low ERβ expression receiving endocrine therapy

**Figure 2:**
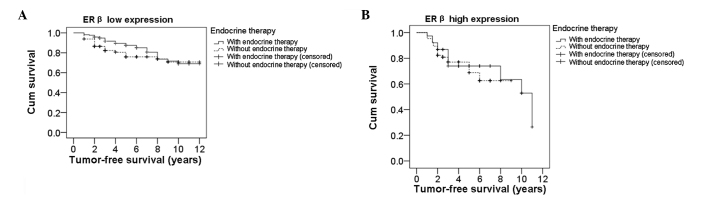
Kaplan-Meier survival analysis of breast cancer patients who received or did not receive endocrine therapy. Differences in survival time were analyzed with the log-rank test. Survival curve of (A) low and (B) high ERβ expression breast cancer patients who received or did not receive endocrine therapy. ER, estrogen receptor.

To determine the effect of ERβ expression on the efficacy of endocrine therapy, survival analysis was performed using the Kaplan-Meier method. Differences in survival time were analyzed with the log-rank test. Firstly, the tumor-free survival times in ERβ low expression patients who received or did not receive endocrine therapy were analyzed. The survival curves of ERβ low expression patients are shown in Fig. 2A. The median tumor-free survival time in patients that received endocrine therapy was 10.11 years, while in patients that did not receive endocrine therapy, the median tumor-free survival time was 9.56 years. Statistically, the difference between these two groups was significant (P=0.038). Next, tumor-free survival times were analyzed in ERβ high expression patients who did or did not undergo endocrine therapy. Fig. 2B shows the survival curves of ERβ high expression patients. In ERβ high expression patients, the median tumor-free survival time of patients that received endocrine therapy was 8.31 years, while the median tumor-free survival time of patients that did not undergo endocrine therapy was 6.85 years. However, there was no statistically significant difference in median tumor-free survival time between these patients (P=0.583). Therefore, these results indicate that high ERβ expression levels in breast cancer patients impair the efficacy of endocrine therapy.

### Patients with low ERβ expression levels have longer a median tumor-free survival time

**Figure 3:**
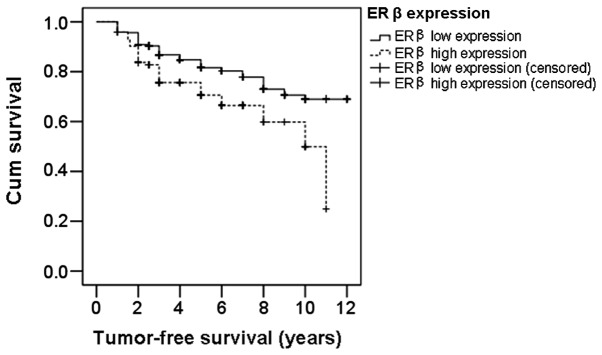
Kaplan-Meier survival analysis of breast cancer patients with low and high ERβ expression levels. Differences in survival time were analyzed with the log-rank test. ER, estrogen receptor.

To further investigate the role of ERβ expression in breast cancer patients, the tumor-free survival times were analyzed using the Kaplan-Meier method and the differences in survival time were analyzed with the log-rank test. The survival curves of ERβ low and high expression patients are shown in Fig. 3. The median tumor-free survival time in patients with low ERβ expression was 9.79 years, while in high ERβ expression patients, it was 8.01 years, which was significantly lower compared with that of the low ERβ expression patients (P=0.002). This result further indicates that patients with high ERβ expression levels have shorter tumor-free survival times and poor prognosis.

### Analysis of prognostic factors for breast cancer

Prognostic factors for breast cancer were analyzed by Cox multivariate analysis. The analyzed factors were ERβ expression, tumor size, pathological grade, lymph node metastasis, chemotherapy, radiotherapy, endocrine therapy, ERα expression and human epidermal growth factor receptor (HER-2) expression. The results are shown in Table II. Independent prognostic factors for breast cancer were identified to be ERβ expression, tumor size, lymph node metastasis, chemotherapy, radiotherapy, endocrine therapy and HER-2 expression (P<0.05). However, pathological grade and ERα expression were not determined to be prognostic factors (P>0.05).

## Discussion

In the present study, tumor-free survival times were compared in breast cancer patients with high and low ERβ expression levels who received or did not receive endocrine therapy. The median tumor-free survival time was 10.11 years in ERβ low expression patients treated with endocrine therapy, while in ERβ low expression patients who did not undergo endocrine therapy, the median tumor-free survival time was 9.56 years. In ERβ high expression patients treated with endocrine therapy, the median tumor-free survival time was 8.31 years, while in ERβ high expression patients without endocrine therapy it was 6.85 years. There was a statistically significant difference (P=0.038) between patients who did or did not receive endocrine therapy when ERβ expression levels were low, whereas there was no significant difference when the ERβ expression levels were high (P=0.583). These results indicate that in ERβ low expression patients, the efficacy of endocrine therapy was significant and the prognosis was better compared with that of the patients who did not receive endocrine therapy. By contrast, in ERβ high expression patients, the efficacy of endocrine therapy was not significant and the prognosis was similar to that of the patients who did not receive endocrine therapy. These results indicate that the prognosis was not improved by endocrine therapy in ERβ high expression patients. In addition, to a certain extent, ERβ high expression may be associated with endocrine resistance. The reason for resistance may result from the binding of ER antagonists with ERβ, which activates the mitogen-activated protein kinase signaling pathway to facilitate the transcription of genes involved in cell proliferation and migration (7).

In addition, ERβ has been reported to have a certain prognostic value (8,9). Chung *et al* (10) used adenovirus vectors to observe the effect of ERβ protein expression on gene transcription in MCF-7 cells. The authors found that ERβ regulated downstream genes, including genes involved in transforming growth factor β signaling, cell cycle, apoptosis and the inhibition of cell proliferation. These observations indicated that ERβ was a poor prognosis factor for carcinogenesis in breast cancer. Jensen *et al* (11) found that ERβ positively expressed breast cancer had a higher histological grade than ERβ negatively expressed breast cancer. In addition, ERβ mRNA expression levels in cancer tissues were upregulated and the prognosis of ERβ and ERα double positive breast cancer patients was poorer compared with ERα single positive patients. In the present study, the median tumor-free survival time for patients with low ERβ expression (9.79 years) was significantly higher compared with that of patients with high ERβ expression (8.01 years; P<0.01). This result was in accordance with previous studies and may be caused by the following two aspects. Firstly, G protein may be activated by estrogen through membrane ERβ, rapidly inhibiting the c-Jun N-terminal kinase pathway and preventing the apoptosis of breast cancer cells (12). Secondly, ERβ may regulate the expression of genes in the Wnt signaling pathway (13). Therefore, ERβ may regulate the proliferation and invasion of breast cancer cells and an imbalance in its expression acts an important indicator for breast cancer recurrence and metastasis.

A previous study (14) found that ERβ expression was associated with axillary lymph node metastasis. Axillary lymph node metastasis is an independent indicator for the treatment and prognosis of breast cancer. Prognosis is relatively poor for breast cancer patients with axillary lymph node metastasis. Multivariate analysis conducted in the present study indicated that ERβ, HER-2, tumor size, lymph node metastasis, postoperative chemotherapy, radiotherapy and endocrine therapy are independent prognostic factors (P<0.05). Positive expression of ERβ and HER-2, larger tumor size, lymph node metastasis, postoperative chemotherapy, radiotherapy and endocrine therapy were risk prognosis factors. This is consistent with previous studies, indicating the positive value of ERβ in prognosis evaluation.

In summary, for the diagnosis and treatment of breast cancer, ERα is measured as a routine pathology test. The 2012 Breast Cancer National Comprehensive Cancer Network treatment guidelines emphasized that adjuvant systemic treatment should be provided according to the expression of ERs. Based on the observations of the present study, it may be hypothesized that ERβ is important for the assessment of postoperative treatment options and prognosis. Combined detection of ERα and ERβ is likely to guide endocrine treatment and prognosis assessment and provide more detailed information for individualized clinical treatment.

## Figures and Tables

**Table I:** Clinical features of the breast cancer patients.

Clinical features	Cases, n (%)
Age, years	
≤49	296 (50.8)
>50	287 (49.2)
Menses	
Menostasis	305 (52.3)
Non-menostasis	278 (47.7)
Tumor size, cm	
≤2	220 (37.7)
>2, ≤5	289 (49.6)
>5	74 (12.7)
Histological grade	
Grade I	108 (18.5)
Grade II	328 (56.3)
Grade III	147 (25.2)
Clinical stage	
Stage 0	193 (33.1)
Stage I	280 (48.0)
Stage II	110 (18.9)
Lymph node metastasis	
Negative	322 (55.2)
Positive	261 (44.8)
ERβ expression	
Negative	460 (78.9)
Positive	123 (21.1)
ERα expression	
Negative	391 (67.1)
Positive	192 (32.9)
HER-2	
Negative	326 (55.9)
Positive	257 (44.1)
Chemotherapy	
Yes	497 (85.2)
No	86 (14.8)
Radiotherapy	
Yes	388 (66.6)
No	195 (33.4)
Endocrine therapy	
Yes	254 (43.6)
No	329 (56.4)

ER, estrogen receptor; HER-2, human epidermal growth factor receptor.

**Table II:** Analysis of prognostic factors for breast cancer by Cox multivariate analysis.

Risk factors	Regression coefficient	Standard error	Wald value	P-value	OR value	95.0% CI
ERβ	0.581	0.212	7.519	0.006^a^	1.787	1.18–2.707
Tumor size						
2–5 cm	0.782	0.285	7.543	0.006^a^	2.187	1.251–3.822
>5 cm	1.162	0.337	11.877	0.001^a^	3.196	1.65–6.188
Pathological grade						
Grade II	0.044	0.281	0.025	0.875	1.045	0.603–1.812
Grade III	0.192	0.309	0.385	0.535	1.212	0.661–2.222
Lymph node metastasis						
1–4 pieces	0.609	0.252	5.829	0.016^a^	1.839	1.121–3.016
5–10 pieces	1.116	0.289	14.902	<0.001^a^	3.053	1.732–5.382
>10 pieces	1.101	0.313	12.361	<0.001^a^	3.006	1.628–5.553
Chemotherapy	1.085	0.231	22.098	<0.001^a^	2.96	1.883–4.653
Radiotherapy	0.556	0.208	7.135	0.008^a^	1.744	1.16–2.623
Endocrine therapy	0.432	0.215	4.024	0.045^a^	1.541	1.010–2.35
ERα	−0.332	0.228	2.118	0.146	0.717	0.459–1.122
HER-2	0.428	0.194	4.871	0.027^a^	1.534	1.049–2.243

a P<0.05.

ER, estrogen receptor; HER-2, human epidermal growth factor receptor; OR, odds ratio; CI, confidence interval.
